# Radiomics Analysis of PET and CT Components of ^18^F-FDG PET/CT Imaging for Prediction of Progression-Free Survival in Advanced High-Grade Serous Ovarian Cancer

**DOI:** 10.3389/fonc.2021.638124

**Published:** 2021-04-13

**Authors:** Xihai Wang, Zaiming Lu

**Affiliations:** Department of Radiology, Shengjing Hospital, China Medical University, Shenyang, China

**Keywords:** high-grade serous ovarian cancer, progression-free survival, radiomics, PET/CT, nomogram

## Abstract

**Objective:**

To investigate radiomics features extracted from PET and CT components of ^18^F-FDG PET/CT images integrating clinical factors and metabolic parameters of PET to predict progression-free survival (PFS) in advanced high-grade serous ovarian cancer (HGSOC).

**Methods:**

A total of 261 patients were finally enrolled in this study and randomly divided into training (n=182) and validation cohorts (n=79). The data of clinical features and metabolic parameters of PET were reviewed from hospital information system(HIS). All volumes of interest (VOIs) of PET/CT images were semi-automatically segmented with a threshold of 42% of maximal standard uptake value (SUVmax) in PET images. A total of 1700 (850×2) radiomics features were separately extracted from PET and CT components of PET/CT images. Then two radiomics signatures (RSs) were constructed by the least absolute shrinkage and selection operator (LASSO) method. The RSs of PET (PET_RS) and CT components(CT_RS) were separately divided into low and high RS groups according to the optimum cutoff value. The potential associations between RSs with PFS were assessed in training and validation cohorts based on the Log-rank test. Clinical features and metabolic parameters of PET images (PET_MP) with P-value <0.05 in univariate and multivariate Cox regression were combined with PET_RS and CT_RS to develop prediction nomograms (Clinical, Clinical+ PET_MP, Clinical+ PET_RS, Clinical+ CT_RS, Clinical+ PET_MP + PET_RS, Clinical+ PET_MP + CT_RS) by using multivariate Cox regression. The concordance index (C-index), calibration curve, and net reclassification improvement (NRI) was applied to evaluate the predictive performance of nomograms in training and validation cohorts.

**Results:**

In univariate Cox regression analysis, six clinical features were significantly associated with PFS. Ten PET radiomics features were selected by LASSO to construct PET_RS, and 1 CT radiomics features to construct CT_RS. PET_RS and CT_RS was significantly associated with PFS both in training (P <0.00 for both RSs) and validation cohorts (P=0.01 for both RSs). Because there was no PET_MP significantly associated with PFS in training cohorts. Only three models were constructed by 4 clinical features with P-value <0.05 in multivariate Cox regression and RSs (Clinical, Clinical+ PET_RS, Clinical+ CT_RS). Clinical+ PET_RS model showed higher prognostic performance than other models in training cohort (C-index=0.70, 95% CI 0.68-0.72) and validation cohort (C-index=0.70, 95% CI 0.66-0.74). Calibration curves of each model for prediction of 1-, 3-year PFS indicated Clinical +PET_RS model showed excellent agreements between estimated and the observed 1-, 3-outcomes. Compared to the basic clinical model, Clinical+ PET_MS model resulted in greater improvement in predictive performance in the validation cohort.

**Conclusion:**

PET_RS can improve diagnostic accuracy and provide complementary prognostic information compared with the use of clinical factors alone or combined with CT_RS. The newly developed radiomics nomogram is an effective tool to predict PFS for patients with advanced HGSOC.

## Introduction

Ovarian carcinoma is the leading cause of gynecologic cancer deaths because the majority of patients are diagnosed with advanced-stage disease (Stages III and IV) according to the International Federation of Gynecology and Obstetrics (FIGO) staging classification (1). HGSOC accounts for up to 70% of epithelial ovarian carcinoma (2, 3). Although most of those women achieve complete remission with cytoreductive surgery and cisplatin based chemotherapy. The median PFS time is only 18 months (4). A significant proportion of patients with advanced HGSOC experience tumor recurrence and progression within 3 years (5). Identification of tumor recurrence and progression in patients with advanced HGSOC after cytoreductive surgery is important since it guides the decisions about personalized treatment and surveillance plans.

^18^F-FDG PET/CT examination can provide more accurate information on preoperative staging and surveillance for detecting recurrent HGSOC (6–9). Compared with CT, ^18^F-FDG PET/CT can identify recurrence earlier because recurrence is characterized by hypermetabolism (9). Previous studies demonstrated conventional PET imaging metrics such as maximum standardized uptake value (SUVmax), metabolic tumor volume (MTV), total lesion glycolysis (TLG) had been reported to be significant prognostic factors for patients with HGSOC (10). However, due to inconsistent result of previous studies, there are some problems with metabolic parameters to predict survival for patients with HGSOC (11–14). Therefore the predictive value of these metabolic metrics to accurately stratify different risk groups seems to be limited (15). More effective indicators are needed to long-term monitor and predict the risk of recurrence and tumor progression.

Radiomics based on high-dimensional quantitative features extracted from different medical imaging modalities can noninvasively quantify tumor heterogeneity and show underlying malignant features (16). On the basis of predictive models based on those radiomics features, clinicians can deliver more personalized medical care about tumor diagnosis, histopathological classification, therapeutic assessment, and prognosis (16, 17). Several studies investigated the role of applying radiomics features extracted from CT images for non-invasive predicting tumor recurrence of HGSOC patients (18–21). The nomogram built by radiomics signatures and clinical factors demonstrated the feasibility of predicting the recurrence of HGSOC (18, 21). However, to our knowledge, study on the establishment and validation of PET/CT radiomics signature and nomogram for predicting PFS in HGSOC patients has not yet been reported. Therefore, in this study we established PET_RS and CT_RS, and hybrid radiomics nomograms integrating RS and clinical factors. In addition, the performances of these hybrid nomograms were compared.

## Materials and Methods

### Patients

This retrospective study was approved by the Medical Ethics Committee of Shengjing Hospital of China Medical University. From January 2013 to December 2017, A total of 363 patients were enrolled in this retrospective study. Inclusion criteria were as follows: (1) patients received cytoreductive surgery and 6-8 cycles of platinum-based chemotherapy; (2) postoperative pathological examination confirmed stage III and IV HGSOC; (3) ^18^F-FDG PET/CT examination was performed before surgery and neoadjuvant chemotherapy (NACT); (4) clinical, pathological, and follow-up information was available. The exclusion criteria included the following: (1) patients received any antitumor therapy before ^18^F-FDG PET/CT scan; (2) patients with other malignancies or other diseases that might affect the radiomics and survival analysis;(3) incomplete clinical-pathological reports;(4) poor image quality or SUVmax<2.5. Finally, 261 patients were enrolled in this study and randomly divided into training (n=182) and validation(n=79) cohorts in a ratio of 7:3. Clinical characteristics including age, FIGO stage, CA125, lymph node metastasis (LNM), volume of ascites, location of primary tumor, residual tumor(>2cm), NACT, and follow-up information were retrieved from the hospital information system. The diameter of primary tumor was acquired by PET/CT images.

### Follow-Up and Clinical Endpoints

The patient was followed up 2-4 months for two years, then 3-6 months for 3 years, then annually after 5 years. Physical exam, CT scan, and serum CA-125 level was used to evaluate recurrence or progression of tumors. The endpoint of this study was tumor recurrence or progression, which was diagnosed by combining clinical symptoms, rising CA-125 levels, and radiological findings. PFS is defined as the time from the end of chemotherapy until tumor progression or the time of last follow-up.

### PET/CT Image Acquisition

All patients underwent a whole-body ^18^F-FDG PET/CT scan on a dedicated PET/CT system (Discovery 690, GE Healthcare, Milwaukee, USA) according to the European Association of Nuclear Medicine (EANM) guidelines within 1 month before any treatment. Patients fasted 6 hours were injected with 161–361 MBq (4.35–9.76 mCi, 150 μCi/kg) ^18^F-FDG. Then the scan was performed after 60 min (59 ± 3 min, range 53–62 min). The 3D ordered subset expectation maximization algorithm (2 iterations and 20 subsets) was used for PET image reconstruction, resulting in voxel sizes of 3.65×3.65×3.27 mm^3^. The field of view (FOV) was 700 mm. The CT scans (80 mA, 120 kV) with matrix sizes of 512×512 were acquired for the attenuation correction method, prior to the PET scan. The PET and CT scans were transferred to workstation to display frame-on-frame fusion images. The PET images (voxel size 3.65 mm, slice thickness 3.27 mm) were then interpolated to the same resolution as CT images (voxel size 0.98 mm, slice thickness 3.27 mm).

### Image Segmentation and Preprocessing

**Figure 1:**
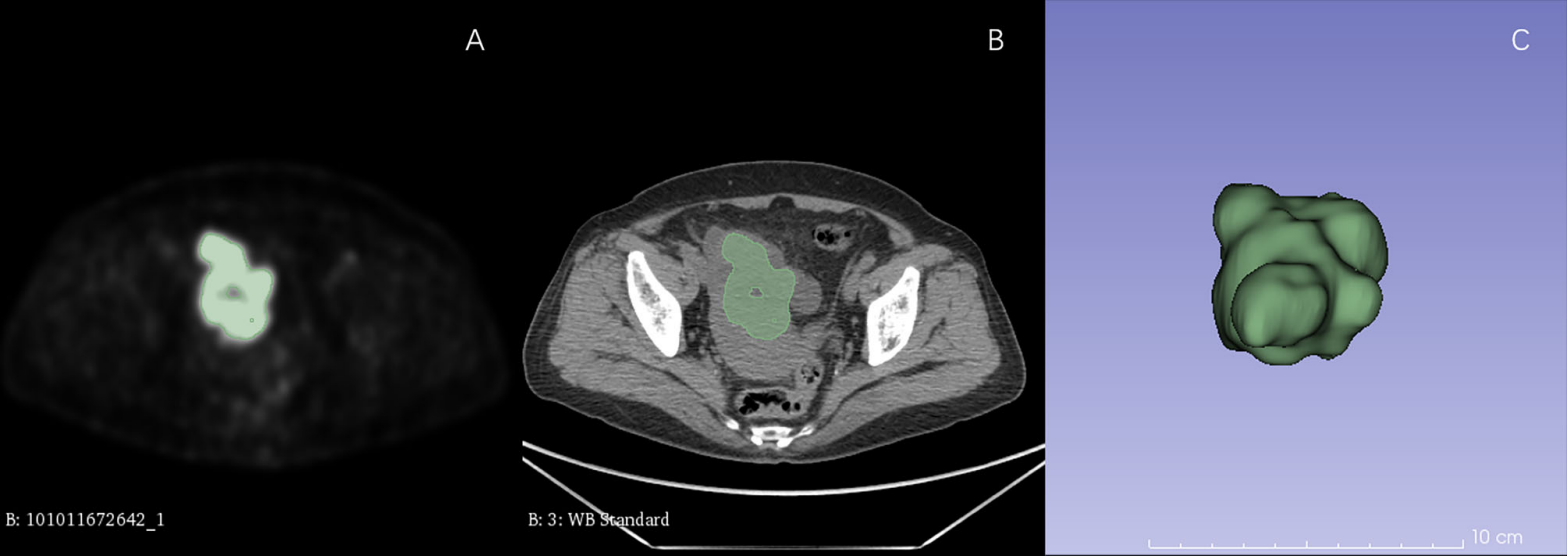
ROI delineation in PET and CT images. Plots **(A, B)** showed ROI delineation in PET and CT images. Plots **(C)** showed the 3D view.

For each patient, all the lesions including primary tumors and distant metastases were identified by two radiologists (with more than 5 years of experience in abdominal imaging) in PET images. Metabolic parameters including SUVmax, SUVpeak, SUVmean, SUVmedian, TLG, MTV were extracted from all lesions. After that, all VOIs of PET images were semiautomatically segmented with a threshold of 42% of SUVmax by 3D slicer (Version 4.81, www.slicer.org). All tumors with SUV > 42% SUVmax were delineated except small lesions with size <1 cm. The VOIs of CT images were delineated according to the VOIs of PET images (**Figure 1**).

### Radiomics Features Extraction and Selection

Extractions of radiomics features from VOIs were performed by using a radiomics extension of 3D Slicer software called SlicerRadomics (V2.10, https://github.com/Radiomics/SlicerRadiomics) (22). We used a fixed bin width to make a histogram and discretized image gray level because PET show a better reproducibility of features when implementing a fixed bin width (23). Finally, 850 radiomics features were extracted from original and 8 derived images obtained by applying Wavelet filters, including 18 first-order features, 13 shape features, 23 gray level cooccurence matrix features, 16 gray level run length matrix features, 16 gray level size zone matrix features, 5 neighboring gray tone difference matrix features, 14 gray level dependence matrix features. All of the radiomics features were separately extracted from VOIs of PET and CT images for each patient.

The univariate analysis based on Cox regression was used to assess the correlation between radiomics features and PFS in training cohort. The features with P-value <0.05 were separately included in the LASSO regression analysis with 5-fold cross-validation for further features selection and RSs calculating. PET_RS and CT_RS of each patient were separately calculated from selected features weighted by their regression coefficients. In the training cohort the optimum cut-off value of each patient for PET_RS and CT_RS was determined by using the time-dependent ROC curve analysis with the highest Youden index, then the patients were divided into high-RS and low-RS groups according to the cutoff values. The potential associations of RSs with PFS were assessed in the training and validation cohorts based on Log-rank test.

### Predictive Model Establishment and Evaluation

The radiomics features of CT and PET with variance close to 0 were deleted. Clinical features and PET metabolic parameters were assessed by univariate analysis based on Cox regression analysis. The features with P-value <0.05 in univariate Cox regression analysis were included in multivariate Cox regression analysis. Only the significant risk features with P-value <0.05 in multivariate Cox regression analysis were used to construct predictive models. Then, multivariate Cox regression models involved different combinations of clinical features, PET metabolic parameters, PET_RS, and CT_RS were build: (1) clinical features alone (denoted as Clinical), (2) combining clinical features and PET metabolic parameters (denoted as Clinical + PET_MP), (3) combining clinical features and PET_RS (denoted as Clinical + PET_RS), (4) combining clinical features and CT_RS (denoted as Clinical + CT_RS), (5) combining clinical features, PET metabolic parameters, and PET_RS (denoted as Clinical + PET_MP + PET_RS), (6) combining clinical features, PET metabolic parameters, and CT_RS (denoted as Clinical + PET_MP + CT_RS). The C-index was used to evaluate the discrimination of models. Then the time-dependent C-index curve analysis was used to evaluate the predictive performance of different models at different time points during follow-up both in training and validation cohorts. Calibration curves were performed to compare the predicted time with actual PFS. In order to evaluate the improvement in prediction performance by adding RSs and PET_MP to the Clinical model, the categorical NRI was calculated in the validation cohort for the first and third year. The patients were classified into three groups based on the probability of tumor progression with cutoffs at 0.30 and 0.60 defining low-, medium-, and high-risk groups. Finally, to provide patients and clinicians with an individualized and easy-to-use postoperative predictive tool for PFS, a radiomics nomogram was constructed on the basis of an optimal model.

### Statistical Analysis

Student t-tests and Mann–Whitney U tests were used for continuous clinical risk factors, Chi-squared tests were applied for categorical variables, and log-rank tests were conducted for PFS to assess the difference between the training and validation cohorts. Univariate and multivariate Cox regression, LASSO-Cox regression analysis, calibration curves plot, C-index, and NRI was performed using R software (version 4.0, http://www.r-project.org). A two-sided p <0.05 indicated a statistically significant difference.

## Results

### Clinical Characteristics

The demographic and clinical characteristics of training and validation cohorts were shown in **Table 1**. There was no significant difference between two cohorts. The PFS of the training cohort was 694 days, 616 days in the validation cohort (P=0.955). Although slightly longer compared with validation cohort, the PFS was not significantly different between two cohorts.

**Table 1:** The demographic and clinical characteristics of HGSOC patients in the training and validation cohorts.

Characteristic	Training cohort (n=182)	Validation cohort (n=79)	P-value
CA125, median (range)	1471 (38.61, 6659.00)	1415 (35.15, 5000)	0.77
Age, mean ± SD, years	55.11 ± 8.90	56.6± 10.31	0.26
NACT			0.66
Yes	55	21	
No	127	58	
Residual tumor			0.99
Yes	49	22	
No	133	57	
Ascites			0.38
<200ml	58	21	
200ml-1000ml	42	15	
>=1000	82	43	
LNM			0.62
Yes	93	37	
No	89	42	
FIGO Stage			1.00
Stage III	123	54	
Stage IV	59	25	
Progression-free survival			0.25
Yes	152	71	
No	30	8	
PFS time	694	616	0.20
Location			0.30
Unilateral	52	17	
Bilateral	130	62	
Diameter, mean ± SD, mm	81.17±33.99	76.1±30.90	0.24

Univariate Cox regression analysis revealed that NACT, residual tumor, ascites, LNM, location, FIGO stage were significantly associated with PFS in the training cohort. But only NACT, location, ascites, and residual tumor was significantly factor associated with PFS in multivariate Cox regression analysis. However, there were no PET_MPs significantly associated with PFS in the training cohort (**Table 2**).

**Table 2:** Univariate and multivariate Cox analysis for PFS in the training and validation cohorts for patients with advanced HGSOC.

Variable	Univariate Cox regression		Multivariate Cox regression	
	HR (95% CI)	P	HR (95% CI)	P
CA125	1.00 (1.00,1.00)	0.08		
Age	1.01 (0.99,1.03)	0.27		
NACT	1.44 (1.03,2.02)	0.04	1.85 (1.25,2.73)	<0.01
Residual tumor	1.96 (1.38,2.79)	<0.01	1.98 (1.34,2.92)	<0.01
Ascites				
<200ml	Reference	Reference		
200ml-1000ml	1.47 (0.95,2.28)	0.09	1.56 (0.99,2.45)	0.05
>=1000	1.89 (1.29,2.75)	<0.01	1.63 (1.10,2.42)	0.02
LNM	1.48 (1.08,2.04)	0.02	1.2 (0.86,1.69)	0.28
Location	1.92 (1.33,2.79)	<0.01	1.76 (1.20,2.59)	<0.01
Diameter	1.00 (0.99,1.00)	0.28		
FIGO Stage	1.43 (1.02,2.01)	0.00	0.9 (0.82,1.71)	0.37
SUVmedian	0.91 (0.83,1.00)	0.06		
TLG	1.00 (1.00,1.00)	0.38		
MTV	1.00 (1.00,1.00)	0.12		
SUVpeak	0.98 (0.95,1.01)	0.27		
SUVmax	0.99 (0.96,1.01)	0.35		
SUVmean	0.92 (0.84,1.01)	0.07		

### Radiomics Signatures Development and Validation

**Figure 2:**
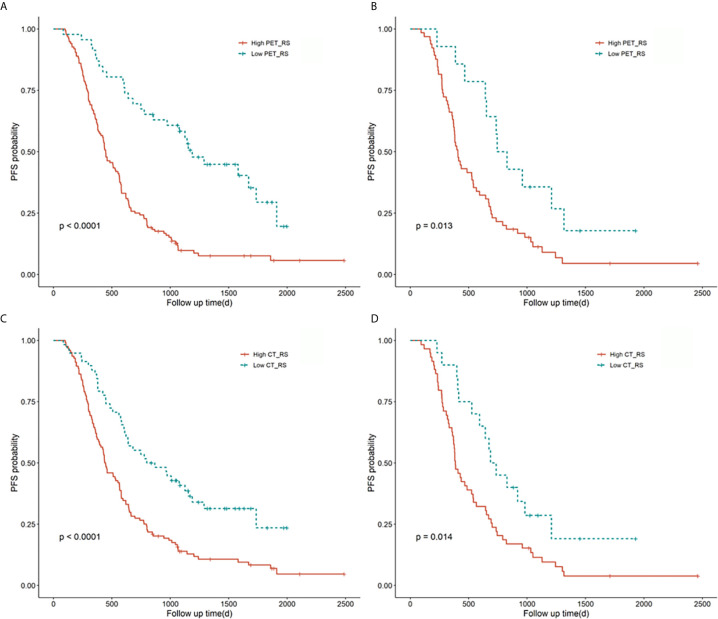
Kaplan-Meier survival curves for PET_RS and CT_RS in the training and validation cohorts. Plots **(A, B)** showed the KM survival curves of PET_RS in the training and validation cohorts. Plots **(C, D)** showed the KM survival curves of CT_RS in the training and validation cohorts.

Ten features were selected from the radiomics feature of PET to construct PET_RS, and 1 features from the radiomics feature of CT to construct CT_RS in the training cohort. RSs were constructed based on the selected features and their corresponding weighting coefficients. The optimal cut-off values for CT_RS and PET_RS were 13.97 and -0.14. There was a significant difference between high and low RS groups both in training and validation cohorts for PET_RS and CT_RS. In the training cohort, p-values of the Log-rank test were both P < 0.01 for PET_RS and CT_RS, and both P=0.01 in the validation cohort. The Kaplan-Meier survival curves were conducted respectively for PET_RS and CT_RS in training and validation cohorts (**Figure 2**).

### Construction of Multiple Prognostic Model and Performance of Different Models

**Figure 3:**
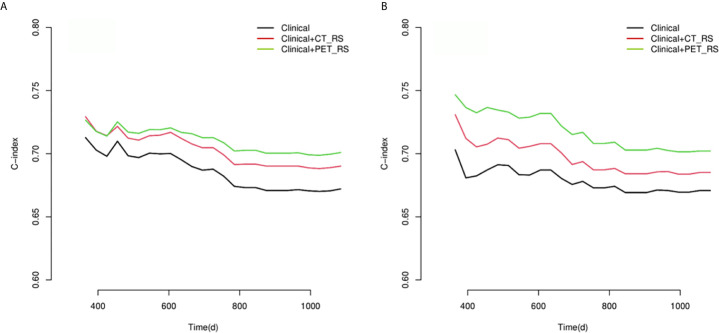
Compare of time-dependent C-index curves of each model for predicting PFS with advanced HGSOC in training **(A)** and validation **(B)** cohorts.

**Figure 4:**
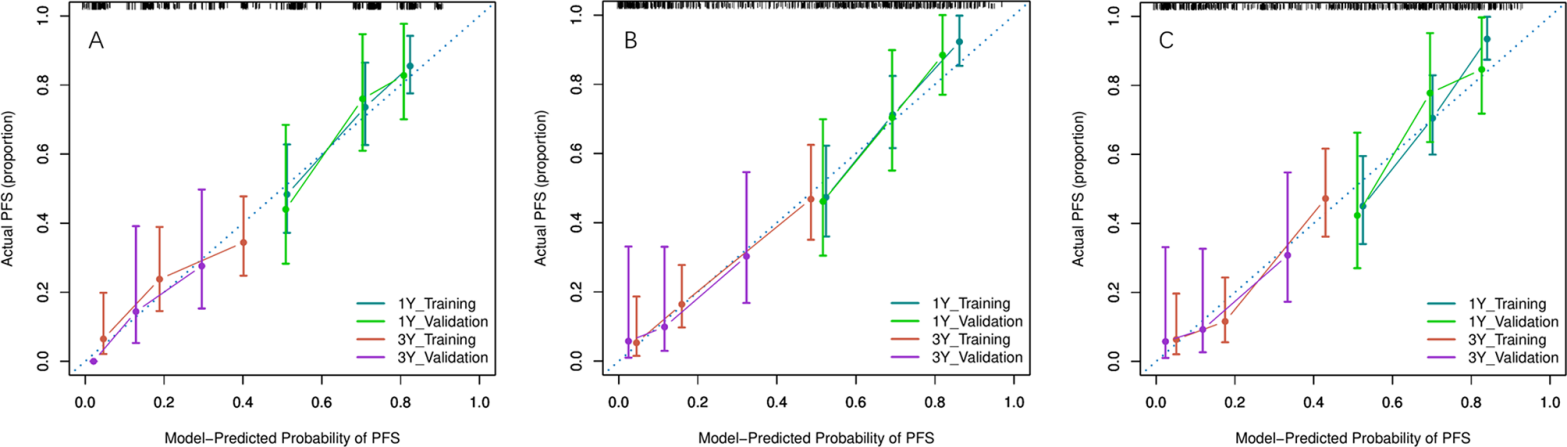
Calibration curves of each model for prediction of 1-, 3-year PFS in the training and validation cohorts. Model-estimated PFS was plotted on the x-axis; the observed PFS was plotted on the y-axis. The diagonal dotted line was a perfect estimation by an ideal model. **(A)** Clinical model, **(B)** Clinical + PET_RS model, **(C)** Clinical + CT_RS model.

Because there were no PET_MPs significantly associated with PFS in the training cohort, only three models were constructed by 4 clinical features with P-value <0.05 in multivariate Cox regression and RSs (Clinical, Clinical+ PET_RS, Clinical+ CT_RS). The C-index of each model was shown on **Table 3**. Clinical+ PET_RS model showed higher prognostic performance than other models in training cohort (C-index=0.70, 95% CI 0.68-0.72) and validation cohort (C-index=0.70, 95%CI 0.66-0.74). The time-dependent C-index curve analysis of each model in training and validation cohorts also indicated similar results (**Figure 3**). Calibration curves of each model for prediction of 1-, 3-year PFS indicated Clinical +PET_RS model showed excellent agreements between estimated and the observed 1-, 3-outcomes (**Figure 4**). Compared to the Clinical model, the Clinical+PET_RS model achieved higher predictive performance improvement than Clinical+CT_RS models in the validation cohort, NRI was 19.33% (95%CI -3.37%,44.23%) for PFS estimation at the first year, and 11.97% (95%CI -6.56%, 29.70%) at the third years (**Table 4**).

**Table 3:** The C-index of each model in the training and validation cohorts.

Model	Training cohort		Validation cohort	
	C-Index	(95% CI)	C-Index	(95% CI)
Clinical	0.67	0.65-0.69	0.67	0.63-0.71
Clinical + PET_RS	0.70	0.68-0.72	0.70	0.66-0.74
Clinical + CT_RS	0.69	0.67-0.71	0.68	0.64-0.72

**Table 4:** NRI in validation cohort for the first year and third year.

Model	Validation cohort(1Y)	Validation cohort(3Y)
	NRI (95%CI)	NRI (95%CI)
Clinical	Reference	Reference
Clinical + CT_RS	3.03 (-15.14, 20.89)	7.05 ( -7.56,23.62)
Clinical + PET_RS	19.33 (-3.37,44.23)	11.97 (-6.56, 29.70)

### Individualized Nomogram Construction and Clinical Use

**Figure 5:**
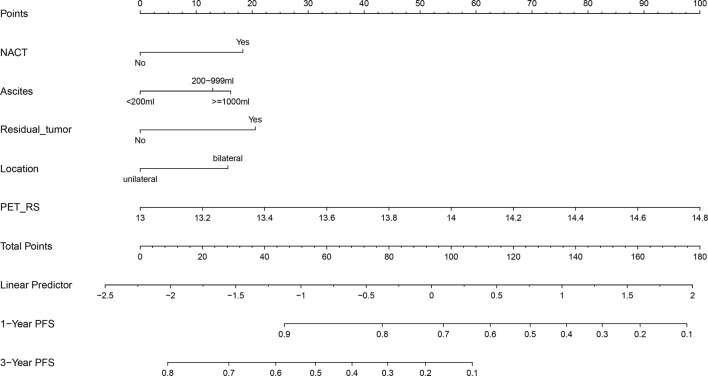
Nomogram based on Clinical + PET_RS model.

Considering that the Clinical + PET_RS model had better discrimination, and calibration in predicting PFS for patients with advanced HGSOC in training and validation cohort, we created a nomogram based on this model, which can visualize proportion of risk factors and prediction result for each patient (**Figure 5**).

## Discussion

Identifying new quantitative imaging markers of PET/CT to improve the accuracy of predicting tumor recurrence and progression is essential for the selection of appropriate treatment and follow up. In this study, we investigated the performance of RSs extracted separately from PET and CT components of PET/CT images integrated with clinical features to predict PFS for patients with advanced HGSOC. Compared with simple use of clinical features, the predictive performance of the model integrated clinical features and RS of PET images were significantly improved both in the training and validation cohorts.

In our study, we only included the patients with advanced HGSOC (FIGO stage III and IV), because there was significant difference between early stage and advanced stage HGSOC in PFS. Besides in our study, some advanced HGSOC patients with high tumor burden received NACT before cytoreductive surgery. We included age, NACT, residual tumor, volume of ascites, LNM, CA125, FIGO stage, location of primary tumor, and diameter of primary tumor as clinical features. Previous study indicated the volume of ascites was an independent predictor of PFS and OS in patients with epithelial ovarian carcinoma (24, 25). Although in previous study the threshold of volume was set to 1500 ml or 2000 ml respectively to classify patients into small- and large-volume ascites groups. We think the patients could be accurately stratified according to small(<200ml), medium (200-999ml), and lager(>1000ml) volume of ascites. AS showed in univariate and multivariate Cox regression analysis, the volume of ascites was the features significantly associated with PFS and included into final model. Previous study built a clinical model involving age, FIGO stage, preoperative CA-125, tumor location,and tumor diameter as features for PFS prediction (18). But in our study, the clinical model only involved clinical features with p-value <0.05 in univariate and multivariate COX regression analysis. Only NACT, residual tumor, ascites, location was included in the final clinical model. Residual tumor, NACT and location of primary tumor was considered to be independent risk factor of PFS for patients with advanced HGSOC in previous study (26–28).

Metabolic parameters of PET images were most frequently used in clinical practice and studies (29, 30). Although as shown in previous meta-analysis, MTV and TLG was potentially useful prognostic markers of PFS and OS in patients with ovarian cancer (13, 14). The prognostic value of metabolic parameters such as SUVmax, SUVmean, SUVmedian, MATV, and TLG for patients with HGSOC remains controversial (8, 11, 12, 15). In our study, Although the P value of SUVmedain and SUVmean was close to 0.05, there was no PET_MP significantly associated with PFS (P<0.05). So we did not include any PET_MP into our model. This might be due to the different cohorts. Previous studies included all subtypes of ovarian cancer regardless of heterogeneity and hindered the subtype-specific significance of PET/CT metabolic parameters. Another possible reason was that the difference of those metabolic parameters of ^18^F-FDG PET/CT in advanced HGSOC was small. Compared with the conventional PET_MP, the radiomics features of PET can reflect more extensive properties of image. PET_RS calculated by lasso regression could directly associate with PFS.

Intratumoral heterogeneity of PET/CT has been proved to be a prognostic predictor for some malignancies these years (10, 31, 32). The radiomics features extracted from PET/CT images allowed us to assess intratumoral and metabolic heterogeneity quantitatively. The relationship between texture-based quantitative features of CT images with residual tumors and survival was revealed in previous studies (33, 34). Textural analysis of CT images can provide added value in evaluating prognosis for patients with HGSOC. The predictive model built in previous study integrating deep learning features extracted from CT achieved good performance (18). The C-index was about 0.7, which is almost equal to our result. Combination of the radiomics feature of F2-Shape/Max3DDiameter with clinical features could significantly improve the AUC for predicting the risk of disease progression within 12 months in ovarian cancer patients (20). Hoverer this result was not validated in other patents. In previous study the AUC of RS constructed by 7 features from CT images to predict 3-year clinical recurrence-free survival was 0.8567 in the training cohort, and 0.8533 in the validation cohort (19). In our study we could forecast the time of tumor progression because the endpoint of our study was tumor progression instead of a fixed interval. However, in our study, the C-index of the CT_RS model was not as high as in the training cohort. A possible explanation for the low predictive power of CT_RS model in the validation cohort was that the VOI outlined in the PET image with a threshold of 42% SUVmax could influence the radiomics features extracted from the CT component of PET/CT image. The intratumoral heterogeneity reflected by CT_RS might decrease. And also, the radiomics features extracted from noncontract CT of PET/CT. Although diagnostic accuracy of CT without contrast media is really poor. The radiomics features of CT without contrast media could also show certain tumor heterogeneity (35–37).The difference between radiomics features of noncontract CT and contrast enhanced CT need to be explored.

To our knowledge, the association between radiomics features of PET images and PFS of HGSOC patients has not been evaluated. In our study, numerous prediction models, incorporating clinical features, CT_RS, and PET_RS in different combinations were built to predict PFS for patients with advanced HGSOC. The Clinical+PET_RS model performed better than other models in the training and validation cohorts. PET_RS included 10 radiomics features extract from original and derived images. The CT_RS was calculated by wavelet. HHH_glszm_GrayLevelNonUniformityNormalized features. This radiomics features measured the variability of gray-level intensity values in CT image array and be related to the PFS. Some studies have shown that the metabolic modifications of PET were more predictive than morphological modifications of CT (35–37). The results of previous and our study indicated the fact that the combination of radiomics features of PET and clinical variables has a more complementary and synergistic effects in predicting PFS.

This study has several limitations. Firstly, this was a single-center study. Although all the PET/CT scans were performed by one PET/CT scanner with standard imaging processes to reduce variance and bias of radiomics features. Further confirmation of the robustness of radiomics features and our predictive model will be needed. Secondly, the VOI s of the tumors was delineated on PET images with 42% SUVmax instead of CT images. The information on anatomical structure and structural heterogeneity may be ignored. Some studies draw ROI manually on fused images (38, 39). There are some problems in manual segmentation in the repeatability and stability of radiomics features. A further study exploring the difference between ROI extract methods will be needed. Thirdly, the average value of radiomics features was computed for all the VOIs including primary and metastatic tumors, which might not be the optimal method. The primary or other metastatic tumors must be investigated to generate optimal case-based image features.

## Conclusions

In conclusion, RSs extracted from the PET and CT components of PET/CT images, quantitatively characterizing intratumoral heterogeneity, were associated with PFS of patients with advanced HGSOC. PET_RS can improve diagnostic accuracy and provide complementary prognostic information compared with the use of clinical parameters alone or combined with CT_RS. The newly developed radiomics nomogram is an effective tool to predict PFS for patients with advanced HGSOC.

## Data Availability Statement

The raw data supporting the conclusions of this article will be made available by the authors, without undue reservation.

## Ethics Statement

This study was approved by the Ethics Review Committee of Shengjing Hospital of China Medical University. Requirements for written informed consent were waived by the committee due to the retrospective nature of the study.

## Author Contributions

ZL contributed conception and design. XW and ZL reviewed information of patients and extracted radiomics features. XW performed the statistical analysis and wrote the first draft of the manuscript. ZL reviewed and revised the manuscript. All authors contributed to the article and approved the submitted version.

## Conflict of Interest

The authors declare that the research was conducted in the absence of any commercial or financial relationships that could be construed as a potential conflict of interest.
