# Supplementary material for: Radiomics Analysis of PET and CT Components of 18F-FDG PET/CT Imaging for Prediction of Progression-Free Survival in Advanced High-Grade Serous Ovarian Cancer
Source: Front Oncol. 2021 Apr 13;11:638124. doi: 10.3389/fonc.2021.638124 (PMC8078590; doi:10.3389/fonc.2021.638124)
Supplement: Supplementary file 1 [file Table_1.docx]

**PET_RS** = 0.00081134149×original_shape_Maximum2DDiameterSlice + 0.00002872616 ×original_shape_MajorAxis

- 0.00246285127 × wavelet.LHL_gldm_LargeDependenceLowGrayLevelEmphasis

+ 1.92613648378 × wavelet.LLH_glrlm_ShortRunLowGrayLevelEmphasis

- 0.91200684287 × wavelet.HLH_glszm_SizeZoneNonUniformityNormalized

+ 0.07051853618 × wavelet.HHH_gldm_DependenceVariance

+ 5.82836167127 × wavelet.HHH_glcm_SumEntropy

+14.20480248376 × wavelet.HHH_glszm_GrayLevelVariance

- 0.00001176271 × wavelet.HHH_glszm_GrayLevelNonUniformityNormalized

+ 1.28846965640 ×wavelet.HHL_glcm_ClusterTendency

**Radiomics features used to calculate PET_RS**

**Maximum 2D diameter (Slice)**

Maximum 2D diameter (Slice) is defined as the largest pairwise Euclidean distance between tumor surface mesh vertices in the row-column (generally the axial) plane.

**Major Axis Length**

This feature yield the largest axis length of the ROI-enclosing ellipsoid and is calculated using the largest principal component λmajorλmajor.The principal component analysis is performed using the physical coordinates of the voxel centers defining the ROI. It therefore takes spacing into account, but does not make use of the shape mesh.

**Large Dependence Low Gray Level Emphasis (LDLGLE)**

Measures the joint distribution of large dependence with lower gray-level values.

**Short Run Low Gray Level Emphasis (SRLGLE)**

SRLGLE measures the joint distribution of shorter run lengths with lower gray-level values.

**Size-Zone Non-Uniformity Normalized (SZNN)**

SZNN measures the variability of size zone volumes throughout the image, with a lower value indicating more homogeneity among zone size volumes in the image. This is the normalized version of the SZN formula.

**Dependence Variance (DV)**

Measures the variance in dependence size in the image.

**Sum Entropy**

Sum Entropy is a sum of neighborhood intensity value differences.
**Gray Level Variance (GLV)**

Measures the variance in grey level in the image.

**Gray Level Non-Uniformity Normalized (GLNN)**

GLNN measures the variability of gray-level intensity values in the image, with a lower value indicating a greater similarity in intensity values. This is the normalized version of the GLN formula.

**Cluster Tendency**

Cluster Tendency is a measure of groupings of voxels with similar gray-level values.

**CT_RS**=-0.380505 × wavelet.HHH_glszm_GrayLevelNonUniformityNormalized

**Radiomics features used to calculate CT_RS**

**Gray Level Non-Uniformity Normalized (GLNN)**

GLNN measures the variability of gray-level intensity values in the image, with a lower value indicating a greater similarity in intensity values. This is the normalized version of the GLN formula.
